# Functional evaluation of DNA repair in human biopsies and their relation to other cellular biomarkers

**DOI:** 10.3389/fgene.2014.00116

**Published:** 2014-05-23

**Authors:** Jana Slyskova, Sabine A. S. Langie, Andrew R. Collins, Pavel Vodicka

**Affiliations:** ^1^Department of Molecular Biology of Cancer, Institute of Experimental Medicine ASCRPrague, Czech Republic; ^2^Institute of Biology and Medical Genetics, First Faculty of Medicine, Charles UniversityPrague, Czech Republic; ^3^Centre for Brain Ageing and Vitality, Institute for Ageing and Health, Human Nutrition Research Centre, Newcastle UniversityNewcastle upon Tyne, UK; ^4^Environmental Risk and Health Unit, Flemish Institute for Technological Research (VITO)Mol, Belgium; ^5^Department of Nutrition, University of OsloOslo, Norway

**Keywords:** *in vitro* comet-based DNA repair assay, base excision repair, nucleotide excision repair, human solid tissue, methodological report

## Abstract

Thousands of DNA lesions are estimated to occur in each cell every day and almost all are recognized and repaired. DNA repair is an essential system that prevents accumulation of mutations which can lead to serious cellular malfunctions. Phenotypic evaluation of DNA repair activity of individuals is a relatively new approach. Methods to assess base and nucleotide excision repair pathways (BER and NER) in peripheral blood cells based on modified comet assay protocols have been widely applied in human epidemiological studies. These provided some interesting observations of individual DNA repair activity being suppressed among cancer patients. However, extension of these results to cancer target tissues requires a different approach. Here we describe the evaluation of BER and NER activities in extracts from deep-frozen colon biopsies using an upgraded version of the *in vitro* comet-based DNA repair assay in which 12 reactions on one microscope slide can be performed. The aim of this report is to provide a detailed, easy-to-follow protocol together with results of optimization experiments. Additionally, results obtained by functional assays were analyzed in the context of other cellular biomarkers, namely single nucleotide polymorphisms and gene expressions. We have shown that measuring DNA repair activity is not easily replaceable by genomic or transcriptomic approaches, but should be applied with the latter techniques in a complementary manner. The ability to measure DNA repair directly in cancer target tissues might finally answer questions about the tissue-specificity of DNA repair processes and their real involvement in the process of carcinogenesis.

## Introduction

The ability of cells to protect against a large variety of DNA disruptions is a vital process for living organisms. Base excision repair (BER) and nucleotide excision repair (NER) belong to the subgroup of DNA repair mechanisms that are active on structurally modified DNA bases. The biological significance of both pathways is highlighted by the well-known association of BER or NER deficiency with the incidence of inherited (Cleaver et al., 2009) and sporadic types of cancer (Slyskova et al., 2012a). Moreover, the individual's BER and NER capacity is expected to have an influence on the response to anti-neoplastic drug treatment (Pallis and Karamouzis, 2010; Lord and Ashworth, 2012). Therefore, being able to screen an individual's repair capacity may represent a step toward risk assessment and individualized cancer therapy.

Our current knowledge of DNA repair indicates that this process involves many genes that have to work in a synchronized and coordinated way. The simultaneous participation of other processes such as DNA damage signaling, cell cycle controls, and maybe even other (un)known genes, makes DNA repair a multigene and multipathway process. There is a body of evidence concerning different levels of DNA repair gene regulation. The majority of DNA repair genes are polymorphic in the human population, with as yet uncharacterized functional consequences (Ricceri et al., 2012). Therefore, DNA sequence analyses cannot be sufficiently informative for predicting DNA repair activity. Gene expression has been shown to be a misleading source of information, because changes in mRNA levels do not necessarily reflect changes in enzyme activity and vice versa (Damia et al., 1998; Vogel et al., 2000; Paz-Elizur et al., 2007; Stevens et al., 2008; Hanova et al., 2011; Slyskova et al., 2012a,b). This is due to extensive post-transcriptional and post-translational modifications and protein–protein interactions that take part in regulating the activity of repair proteins (Fan and Wilson, 2005; Hu and Gatti, 2011; Nouspikel, 2011). Moreover, DNA repair is a multifactorial process that is modulated not exclusively by genetic background, but, to a certain extent, might be regulated by environmental and lifestyle factors (Wu et al., 2006; Collins et al., 2012). Measuring the true phenotypic endpoint seems in this context to be the most informative, straightforward, and perhaps the most reliable way of characterizing the DNA repair processes.

Both excision pathways follow a common pattern: recognition of the DNA lesion, excision of the damage, and resynthesis of the removed sequence. Transient strand breaks (SBs) are generated as intermediates during both repair pathways, which make BER and NER easily measurable on the functional level by methods based on the comet assay. This assay is a sensitive technique for quantification of SBs in DNA which is applicable not only to measure basal DNA damage, but also in a modified form for measuring DNA repair incision activity. So far, human blood has been used in the majority of studies examining DNA repair activity, since blood is usually the only tissue that can be sampled from healthy subjects. Methods for evaluation of BER and NER from peripheral blood cells are currently well-established (Collins et al., 2001; Langie et al., 2006). However, often it is important to consider the level of DNA repair in particular organs, especially when analysing DNA repair activity in association with tissue-specific diseases. Although it might be logistically complicated in most cases, from cancer patients, there is still a possibility to obtain not only peripheral blood, but also surgically resected normal or tumor tissue. Langie et al. (2010, 2011) have published protocols modified for assessment of DNA repair activity in animal solid tissues; however, a methodological approach for its evaluation in human biopsies has not yet been optimized. Moreover, despite the undeniable biological significance of DNA repair, DNA repair activity is still not routinely included as a biomarker in human biomonitoring studies. This is partially due to the fact that it is a relatively laborious method, especially when large numbers of samples are analyzed.

This text presents a detailed comet assay-based protocol for measuring BER- and NER-specific incision activity *in vitro* from deep-frozen human solid tissues, covering all its optimization steps. The protocol has been recently applied for the first time on colorectal cancer biopsies (Slyskova et al., 2012b). In order to increase the applicability of this approach to large-scale epidemiological studies, the 12-minigel format (12 agarose minigels per microscopic slide) instead of the conventional format (one or two large agarose gels per slide) has been applied. In addition, the relationship between the detected DNA repair activity and other biomarkers (single nucleotide variants in and expression of DNA repair genes) routinely measured in human biomonitoring studies is also discussed.

## Materials and methods

### Study population and collection of biological specimen

The study was conducted on colorectal tissues collected from 70 CRC patients at the surgical resection of the tumor. Patients were recruited between 2009 and 2011 in Thomayer Hospital (Prague), General University Hospital (Prague), and Teaching Hospital and Medical School of Charles University (Pilsen). All patients gave informed consent. Ethics approval was granted by appropriate committees of the three hospitals. The group of patients included 53 men and 17 women with a mean age of 66.2 ± 10.6 years. The clinical stage of patients at diagnosis was classified according to the tumor-node-metastasis (TNM) system. Seven patients were diagnosed with TNM stage I (10%), 29 as stage II (41.4%), 15 as stage III (21.4%), and 19 as stage IV (27.2%). All patients had adenocarcinomas; 44 patients had tumor localized in the colon (62.9%) and 26 in the rectum (37.1%). In 12 (17.2%) patients, tumors were of well-differentiated grade, in 47 (67.1%) moderately differentiated and in 11 patients (15.7%) poorly differentiated. Eleven rectal cancer patients (15.7%) received neoadjuvant therapy prior to surgery. Tumor tissue and adjacent healthy colon/rectal tissue (5–10 cm distant from the tumor) were resected from all patients. Colon biopsies were briefly washed in PBS and snap frozen immediately after the resection and further stored at −80°C. Prior to tissue processing, histological analysis was carried out to assess the proportion of tumor cells in tumor tissues and to rule out the presence of neoplastic cells in the normal mucosal tissues. The cut-off point was set to 80% of tumor or normal cells in the sample, respectively. Samples were embedded in optimal cutting temperature compound (Sakura Finetek), and cut with a Leica CM 1850 cryostat. Five μm thick serial sections were fixed in 90% ethanol on microscope slides and stained with 1% cresyl violet acetate (Sigma-Aldrich), dehydrated with ethanol, dried, and inspected using a Leica DM6000 microscope (Leica). Due to various logistical reasons, not all patients could be analyzed for all the studied parameters. Therefore, each particular analysis is further specified for actual number of cases for whom analysis was carried out.

### *In vitro* comet-based DNA repair assay

#### Principle of the assay

Protein extracts isolated from human tissues were incubated with substrate DNA in the form of nucleoids, containing artificially induced lesions known to be repaired specifically by either the BER or NER pathway. The photosensitizer Ro 19-8022, in the interaction with visible light, gives rise to oxidative DNA damage (predominantly to 8-oxoguanines; 8-oxoG) that are specifically recognized by the BER machinery. Alternatively, nucleoids containing ultraviolet light (UV)-induced cyclobutane pyrimidine dimers (CPD) and 6-4 photoproducts represent the substrate for the NER pathway. The level of induced lesions can be detected by enzymes of bacterial or viral origin, which serve as positive controls in each experiment. Two types of enzymes were used, formamidopyrimidine DNA glycosylase (Fpg) as a prokaryotic analog of human OGG1 that recognizes oxidized purines, and T4 Endonuclease V (Endo V) produced by T4 bacteriophage, recognizing UV-induced CPD. In addition, each experiment included a negative control, namely lesion-containing substrate DNA incubated with reaction buffer to assess the background damage together with buffer-induced damage. Furthermore, each protein extract was measured for (i) specific repair activity (i.e., extract incubated with lesion-containing DNA) and (ii) non-specific endonuclease activity (i.e., specificity control; extract incubated with lesion-free DNA). To be able to record only specific activity of repair proteins, the non-specific endonuclease activity of the protein extract was subtracted. The frequency of DNA SBs, generated during incision of lesions, reflects the DNA repair activity of the extract.

#### Substrate DNA

In this protocol, the cellular source of substrate DNA consisted of peripheral blood mononuclear cells (PBMC) and human-derived lymphoblastoid cells (TK6), though in principle any other mammalian cells in suspension could be used. Cells should be controlled for low basal level of SBs (ideally not higher than 10% DNA in tail) and such was the case in this study. PBMC were separated on Histopaque-1077 (Sigma-Aldrich), counted, evaluated by trypan blue exclusion and suspended in ice-cold PBS. TK6 cells were grown in RPMI medium supplemented with 10% heat-inactivated fetal calf serum, 0.2 mg/mL sodium pyruvate, 2 mM L-glutamine, 100 U/mL penicillin, and 0.1 mg/mL streptomycin (Sigma-Aldrich). Cells were counted and suspended in ice-cold PBS. For BER, PBMC were treated with 2μM Ro 19-8022 (Hoffmann-La Roche) for 5 min, and irradiated on ice at 33 cm distance from a 500 W halogen lamp. For NER, TK6 cells were irradiated with 5 Jm^−2^ of UVC (50 s at 0.1 Jm^−2^s^−1^). Lesion-free PBMC and TK6 cells were prepared in parallel. Cells were aliquoted at 5 × 10^5^ in 0.5 mL of freezing medium (RPMI 1640, 20% fetal bovine serum, 0.2% antibiotics, 10% DMSO, Sigma-Aldrich) and frozen slowly to −80°C. Before each experiment, cells were thawed by adding 1 mL of cold PBS, spun at 400 g, 5 min, 4°C, and suspended in 400 μL of PBS. An 80 μL aliquot of the cell extract was mixed with 260 μL of 1% LMP agarose to reach the desired concentration of cells (~300–600 cells per minigel). Using a multi-dispensing pipette, twelve 5 μL minigels of cells in agarose were placed on each microscope slide. Cells embedded in agarose underwent lysis for 1 h in 2.5 M NaCl, 100 mM EDTA, 10 mM Tris, 250 mM NaOH, 1% Triton X-100, pH 10. Before incubation with protein extracts, slides were washed twice for 5 min with buffer B (45 mM HEPES, 0.25 mM EDTA, 0.3 mg/mL BSA, 2% glycerol, pH 7.8) and placed in incubation chambers (Severn Biotech) (Shaposhnikov et al., 2010).

#### Protein extracts preparation

Tissue resections were weighed and ground while frozen, and 30–50 mg aliquots were stored at −80°C. For extraction, a volume of 100 μL of buffer A (45 mM HEPES, 0.4 M KCl, 1 mM EDTA, 0.1 mM DTT, 10% glycerol, pH 7.8) was added to every 50 mg of ground tissue. Samples were vortexed, snap frozen, and 30 μL of 1% Triton X-100 in buffer A added per 100 μL. Protein concentration was measured by a fluorescamine assay (Sigma-Aldrich), on a NanoDrop 3300 (Thermo Scientific). Undiluted extracts were kept at −80°C. Before the incubation reaction, on the day of use, extracts were diluted to a protein concentration of 3 mg/mL in buffer A in a final volume of 50 μL and mixed with 4 volumes of buffer B.

#### BER-specific reaction

A 30 μL aliquot of extract was added to each minigel in the incubation chamber. Each extract was incubated with Ro-treated as well as non-treated PBMC (used for background subtraction). Incubation time was 20 min, at 37°C in a humid environment. Fpg was used as a positive control. For a negative control substrate DNA was incubated with buffer A + buffer B in a 1:4 ratio. Each experimental point was performed in duplicate. In optimization experiments, PARP inhibitor ABT-888 (Selleckchem) was added to the extract at a concentration of 5 μM to test the effect of inhibiting the post-incision phase of BER.

#### NER-specific reaction

For the NER-specific assay, the protein extract was enriched with adenosine-5′-triphosphate at a final concentration of 2.5 mM. A 30 μL aliquot of extract was added to each minigel in the incubation chamber. Each extract was in parallel incubated for 30 min with UV-treated and non-treated TK6 cells (used for background subtraction). UV substrate incubated with Endo V was used as positive control and 1:4 buffer A + buffer B as negative control. In optimization experiments, aphidicolin (DNA polymerase delta inhibitor; Sigma-Aldrich) at a concentration of 2.5 μM was added to the extract to test the effect of DNA resynthesis inhibition.

#### Single cell gel electrophoresis

After the incubation period, the protocol followed was the same as previously described for the comet assay (Olive and Banath, 2006). In brief: slides were treated for 20 min under alkaline conditions (0.3 M NaOH, 1 mM EDTA, pH 12) to allow DNA denaturation and subsequently electrophoresed for 20 min at 1.3 V/cm. Washing followed, with PBS, then H_2_O and finally ethanol, each for 10 min. Slides were stained with SYBRGold (Invitrogen) at the concentration recommended by the manufacturer in a bath at 4°C with agitation. After 40 min, SYBRGold solution was removed and the slides rinsed twice with water and left to dry at room temperature. On the day of analysis gels were hydrated by adding a drop of water on top of each minigel and covered with a coverslip. The comets were evaluated by visual scoring performed exclusively by one person (Azqueta et al., 2011). Comets were analyzed by a Nikon fluorescence microscope using 5 classes of comets from class 0 (undamaged, no discernible tail) to class 4 (almost all DNA in tail, insignificant head). Hundred comets were selected at random for each sample (50 comets per duplicate gel), so the overall score from one sample ranged from 0 to 400 arbitrary units. Final DNA repair activity was calculated as the difference between scores for treated substrate incubated with extract and non-treated substrate incubated with extract. (Visual scoring was preferred only because of technical problems with image analysis software at the time of the study; however, scoring with computerized software is equally recommended).

### Genotyping

Single nucleotide polymorphisms (SNPs) were selected according to their (i) location in a gene involved in a pre-incision complex of BER or NER whose activity is detectable by DNA repair assays, (ii) minor allelic frequency >5%, and (iii) predicted damaging or deleterious effect on protein function by SIFT or PolyPhen algorithms (Xi et al., 2004). DNA was isolated from total blood by the phenol-chloroform method. SNPs were detected by TaqMan^®^ SNP Genotyping Assays based on allele-specific TaqMan^®^ MGB probes plus PCR primers and analyzed on Applied Biosystems 96-well real-time PCR instrumentation (Life Technologies). Functional SNPs in BER genes were represented by *OGG1* Ser326Cys (rs 1052133). Selected SNPs within NER genes involved *XPA* G23A (rs 1800975), *XPC* Ala499Val (rs 2228000) and Lys939Gln (rs 2228001), *XPD* Lys751Gln (rs 13181), *XPG* Asn1104His (rs 17655) and *XPF* Arg415Gln (rs 1800067).

### Reverse transcription qPCR

Tissue samples were homogenized in the MagNA Lyser (Hoffmann-La Roche). AllPrep DNA/RNA mini kit (Qiagen) was used to isolate nucleic acids. Total RNA was measured on ASP-3700 Spectrophotometer (Avans-Biotechnology) for quantity and OD260/280 ratio. RNA integrity number (RIN) was checked using Agilent Bioanalyzer 2100, with RNA 6000 Nano Assay (Agilent Technologies). cDNA was synthesized from 500 ng of RNA using a RevertAidTM First strand cDNA synthesis kit (Thermo Scientific) using random hexamers and following manufacturer's instructions. cDNA was diluted to 10 ng/μL and preamplified for 18 cycles on a CFX96 Real Time PCR Instrument (Biorad) according to the manufacturer's protocol. qPCR was performed using the high-throughput platform BioMark™ HD System (Fluidigm). Ten μL of reaction mix contained 1 μL of 20× diluted preamplified cDNA, 2.5 μL of Taqman Universal Mastermix II without UNG (Life Technologies), 5 μL of primer/probe assays with Perfect Probe™ (Primer Design) at a final concentration of 300 nM, 2.5 μL of 2× Assay loading reagent and 0.25 μL of 20× GE sample loading reagent (Fluidigm) and 1.25 μL of water. Cycling conditions for qPCR were: 95°C for 10 min, 45 cycles of 95°C for 15 s and 50°C for 60 s. *TOP1* and *18S rRNA* were reference genes selected from a geNorm™ reference genes selection kit (Primer Design) by Normfinder algorithm (GenEx Enterprise software). Data were collected from one 48 × 48 array. Data were normalized to reference genes, converted to relative quantities and transformed to log2 scale.

### Statistical analysis

Statistical analysis was performed by SPSS Statistics 18 (IBM) and by GenEx Enterprise (MultiD) softwares. The distribution of investigated parameters was controlled by Kolmogorov–Smirnov test. Expression data were logarithmically transformed to achieve a normal distribution. Two-tailed *T*-test or ANOVA for differences between groups for normally distributed data was employed and correlations determined by a Pearson's test. When data were not distributed accordingly to a Gaussian curve, non-parametric tests of Kruskal–Wallis, Mann–Whitney or Spearman's correlation coefficient were used. All statistical tests were performed at a 95% confidence level.

## Results and discussion

### Optimization of the BER- and NER-specific assays

#### An advanced medium-throughput 12-minigel format

**Figure 1:**
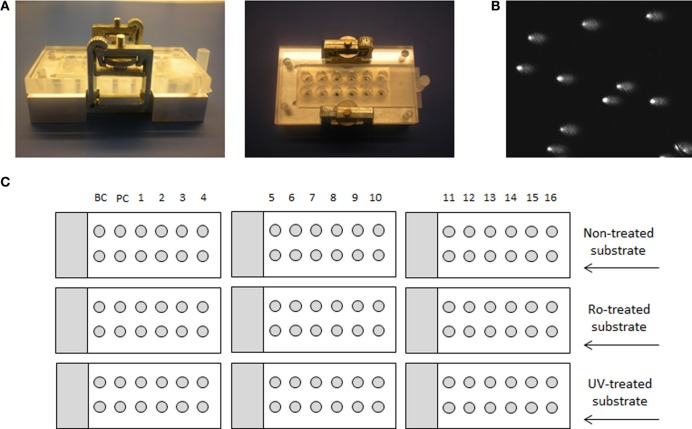
**Medium-throughput comet assay format and layout of the experiment. (A)** Device for 12-minigel format, **(B)** image of comets, and **(C)** a schematic example of an experiment, using 16 different extracts, background control with buffer only (BC) and a positive control with specific enzyme (PC). The experimental layout with 9 microscope slides is applicable only if the same substrate cell-type is used in each assay, so that the same non-treated cells can be used for both.

In order to be able to process a larger number of samples and to suppress the effect of inter-experimental variability, we have utilized the 12-minigel format that was introduced by Shaposhnikov et al. (2010) and is demonstrated in Figure 1. The comparability of the new 12-minigel approach (12 minigels of 5 μl agarose per slide) with the conventional 2-gel format (2 large gels of 70 μl agarose per slide) was tested by Azqueta et al. (2012). Therefore, we have directly optimized the BER- and NER-repair assays for a 12-minigel format, without any additional testing.

The inter-experimental variability given by the 12-minigel format was low, with the coefficient of variation between 7 independent experiments being 7.7% for both BER and NER (calculated from the negative and positive control, data not shown). This suggests that only up to 8% of variability might be attributed to inter-experimental variations. The 12-minigel format, with its 6-times higher yield of analyzed samples per microscopic slide, significantly increases the applicability of repair assays to human epidemiological studies. Sixteen samples can be optimally run for both assays in one experiment, using only 9 microscopic slides and 9 incubation chambers (see scheme displayed in Figure 1). This capacity is not limited by the dimensions of the electrophoretic tank, as is usually the case with the 2-gel format. Another advantage is that the new format requires considerably lower numbers of substrate cells. Instead of ~3000 substrate cells per gel, the 12-minigel format requires only a tenth of this quantity.

#### Precision of the assays

**Figure 2:**
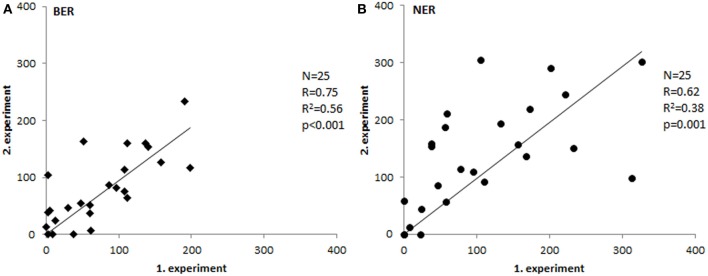
**Inter-experimental variability**. Comparison of tissue extract activities measured in two separate experiments (Spearman's correlation coefficient). Paired *T*-test *p*-values were 0.58 for BER **(A)** and 0.1 for NER **(B)**.

To test repeatability of the assays, we have measured 25 samples in two independent experiments and compared the results. As shown in Figure 2, the inter-experimental variation is negligible and both assays are repeatable with high reliability (*p* ≤ 0.001).

**Figure 3:**
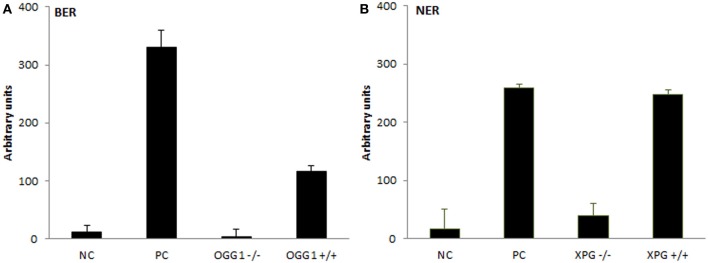
**Testing the extracts from DNA repair gene knock-out cells**. Measurement of BER and NER activities in knock-out cells and wild-type control cells of the same origin. For **(A)** BER assay extracts were isolated from OGG1^−/−^ and OGG1^+/+^ mice livers; negative control (NC) represents incubation with buffer only and positive control (PC) incubation with Fpg. For **(B)** NER assay extracts were isolated from XPG^−/−^ and XPG^+/+^ hamster ovarian cancer cells CHO AA8, and NC and PC represent incubation with buffer and Endo V, respectively. Data represent means ± *SD* of duplicate measurements.

The assays were subsequently tested for the ability to recognize protein extracts deficient in the incision step of repair. For that purpose, extracts from OGG1- and XPG-deficient cells were isolated and their activity compared with the extracts isolated from cells of the same origin but expressing both genes. Figure 3 presents observed results in comparison to positive and negative controls, as described in detail in *BER*- and *NER-specific reaction* sections. While protein extracts from wild-type cells showed activity significantly higher than activity measured for knock-out cells (BER: *p* = 0.007, NER: *p* = 0.019), the low activity of knock-out cells was not different from the unspecific activity of buffer only (BER: *p* = 0.44, NER: *p* = 0.39). Both assays confirmed a sensitivity to distinguish biological variability.

#### Dependence of protein extract activity on protein concentration

**Figure 4:**
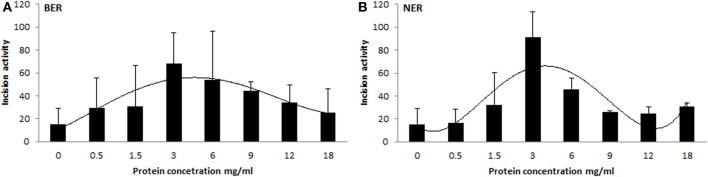
**Relationship between protein concentration of extract and its activity**. Different protein concentrations of tissue extracts plotted against their BER **(A)** and NER **(B)** activities. Each experimental point represents mean ± *SD* of duplicate measurements.

We prepared tissue extract dilutions in the range 0 to 18 mg/mL protein content and measured the corresponding activity. Surprisingly, the relation between protein concentration and activity of the extract was not (log-)linear (higher protein amount corresponding with higher activity), but instead exhibited an increase of incision activity reaching its maximum at a protein concentration of 3 mg/mL, followed by a drop of activity with further increasing content of proteins (Figure 4). At the concentration point of 3 mg/mL the ratio between lesion-specific activity and non-specific endonuclease activity of the extract was the highest and in favor of the former. Another confounder would be represented by the ratio between protein amount and accessibility of DNA lesions. Too high protein concentration leads to saturation of the reaction. However, protein concentration optimal for the assays was shown to be tissue-specific, as studied on animal tissues (Langie et al., 2010, 2011), and therefore the concentration set by us is not generally applicable. The optimal concentration should be tested by each user of the assay on particular biological samples. Time of incubation is also a variable that can be recommended, but anyway should be pre-tested on each particular substrate DNA with specific extracts.

#### Do intermediate SBs reflect the incision activity?

**Figure 5:**
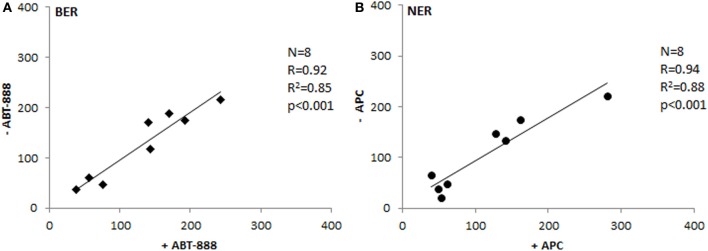
**Testing the inhibition of post-incision phase**. Comparison of BER **(A)** and NER **(B)** activities between extracts treated with inhibitors of polymerization and the same extracts not suppressed for the polymerization activity (Spearman's correlation coefficient). ABT-888, inhibitor of Poly-(ADP)-ribose polymerase; APC, aphidicolin - inhibitor of DNA polymerase delta.

To test the postulate that SBs measured in the assays are generated specifically by the incision activity of the protein extract, we have used specific agents to inhibit post-incision phase of the repair processes. ABT-888 is a well-known inhibitor of the Poly-(ADP)-ribose polymerase (PARP) and has therefore been used in the BER assay. The post-incision NER phase was blocked by aphidicolin (APC), inhibiting the function of polymerase delta. On adding the specific inhibitors, all SBs generated by incision activity are expected to remain “open.” As Figure 5 documents, the tissue extracts correspond in 85–88% of their activities irrespective of the presence of the inhibitors. Thus, BER and NER assays detect specifically the incision step of the whole repair process, which is regarded the rate-limiting step (Collins, 1987; Shivji et al., 1992). It involves proteins that are rather active in the repair of DNA damage, unlike post-incision complexes that take part also in the replication or transcription of DNA. The extract is not able to perform the synthetic stages of repair, unless deoxyribonucleotides, and ATP are provided (Collins et al., 1994).

### DNA repair capacity in relation to other cellular biomarkers

#### Genotype–phenotype interactions

Ro-induced oxidative damage is mainly represented by 8-oxoG. There are several enzymes known to be specialized for this particular lesion; however the 8-oxoguanine DNA glycosylase (OGG1) is the primary enzyme recognizing and incising this lesion. Among others, NEIL1 and NEIL2 have marginal activity in repair of this lesion, NTH1 repairs free 8-oxoG and MUTYH recognizes adenine already mispaired with 8-oxoG. Therefore, BER activity, as measured *in vitro* in our assay toward Ro-induced lesions, is mainly reflecting the activity of the BER glycosylase OGG1. In contrast to BER, NER enzymes work in large complexes and the minimal requirement for the incision comprises at least 20 proteins.

The majority of BER and NER genes are polymorphic in the population, and over 50% of them have functionally relevant amino acid changes. By applying SIFT or PolyPhen algorithms, several SNPs are predicted to be possibly damaging, damaging, or deleterious, by means of protein function (Xi et al., 2004) and these *in silico* characterizations are also supported by a range of epidemiological and *in vitro* studies. In this study, functional SNPs in BER genes were represented by the commonly studied *OGG1* Ser326Cys, while NER genes were represented by *XPA* G23A, *XPC* Ala499Val and Lys939Gln, *XPD* Lys751Gln, *XPG* Asn1104His and *XPF* Arg415Gln. All of these potentially functional SNPs were genotyped in the cohort of 68 individuals and their effects on BER and NER activity of colorectal tissues were studied. None of the studied SNPs showed any direct association with DNA repair activity in either healthy or tumor tissues, except for *XPA* 23A allele that was associated with lower BER in tumor tissues only (Table 1). An association of *XPA* G23A genotype with BER activity in PBMC was observed by Dusinska et al. (2006), although in a relationship opposite to that found by us. Conflicting findings were obtained from studies with Xpa-deficient mice, where XPA seems not to play an important role in oxidative DNA damage repair (Melis et al., 2013). We are aware of low statistical power and risk of type 2 error due to the low number of individuals carrying variant alleles. Nonetheless, reports on genetic variability in relation to DNA repair activity of target tissue (i.e., tissue other than blood) were missing until now.

**Table 1:** **Influence of single nucleotide polymorphisms in BER and NER genes on the BER and NER incision activity (Comparison of medians by Kruskal–Wallis or Mann–Whitney *U*-tests)**.

**SNP**	**Genotype**	***N***	**Healthy tissue (*N* = 68)**				**Tumor tissue (*N* = 68)**			
			**BER median (quartiles)**	***p*-value**	**NER median (quartiles)**	***p*-value**	**BER median (quartiles)**	***p*-value**	**NER median (quartiles)**	***p*-value**
*OGG1* Ser326Cys (rs1052133)	GG	41	15.3 (9.2–20.8)		12.5 (8.0–23.5)		16.9 (10.8–23.8)		19.1 (14.1–27.9)	
	GC	26	14.8 (8.2–23.5)		14.2 (7.3–18.6)		22.3 (12.8–27.6)		18.3 (7.9–21.2)	
	CC	1	6.2	0.49	15.5	0.79	5.6	0.056	20.4	0.22
	GC + CC	27	14.7 (7.2–23.2)	0.87	14.5 (8.0–18.5)	0.54	22.2 (12.3–27.6)	0.072	18.5 (8.3–21.2)	0.09
*XPA* G23A (rs1800975)	GG	23	16.1 (10.7–21.6)		14.9 (9.3–20.4)		22.4 (16.7–27.0)		18.7 (16.1–25.0)	
	GA	33	14.5 (8.6–20.2)		11.9 (5.7–23.5)		16.3 (11.2–23.8)		19.1 (8.7–27.2)	
	AA	12	10.4 (3.4–22.5)	0.50	18.9 (11.2–21.3)	0.36	13.9 (1.9–25.3)	0.056	18.9 (10.5–30.0)	0.87
	GA + AA	45	14.1 (7.0–20.7)	0.27	12.5 (7.8–22.4)	0.79	16.3 (10.3–23.8)	**0.018**	19.1 (9.4–27.2)	0.6
*XPC* Ala499Val (rs2228000)	GG	34	15.3 (8.7–20.5)		14.8 (8.8–24.0)		17.1 (11.2–26.0)		19.2 (11.0–28.9)	
	GA	26	14.7 (9.1–22.0)		14.2 (5.5–18.9)		21.5 (13.8–25.3)		18.4 (11.7–24.5)	
	AA	8	15.6 (6.0–26.3)	0.92	12.2 (6.5–18.8)	0.76	13.6 (1.2–23.0)	0.34	18.9 (9.3–34.4)	0.92
	GA + AA	34	14.7 (8.5–23.4)	0.87	13.7 (5.8–18.9)	0.46	18.7 (12.0–24.7)	0.82	18.4 (10.5–25.7)	0.74
*XPC* Lys939Gln (rs2228001)	TT	30	14.1 (5.9–21.2)		13.7 (8.2–20.4)		18.7 (9.0–24.7)		18.3 (10.5–24.5)	
	TG	28	15.0 (10.0–22.4)		14.6 (3.3–23.7)		17.1 (12.3–25.4)		18.4 (10.5–31.5)	
	GG	10	16.5 (9.2–22.0)	0.42	13.4 (6.7–17.9)	0.92	19.5 (11.9–27.1)	0.73	19.2 (14.2–23.7)	0.85
	TG + GG	38	15.9 (9.6–21.2)	0.25	14.6 (6.7–21.6)	0.82	17.4 (12.3–25.6)	0.56	18.9 (12.5–28.9)	0.57
*XPD* Lys751Gln (rs13181)	GG	28	15.6 (9.0–21.5)		14.5 (8.5–24.4)		18.0 (12.6–24.1)		17.1 (9.3–24.8)	
	GT	29	14.5 (7.7–24.0)		13.9 (8.1–20.9)		21.8 (11.8–26.9)		20.8 (14.6–32.1)	
	TT	11	14.9 (12.3–16.3)	0.93	13.5 (5.2–18.9)	0.84	12.5 (5.6–22.2)	0.38	17.5 (13.9–20.4)	0.19
	GT + TT	40	14.7 (8.6–18.3)	0.76	13.7 (7.7–20.3)	0.67	17.2 (11.2–26.5)	0.89	19.6 (14.0–28.1)	0.15
*XPF* Arg415Gln (rs1800067)	GG	59	15.3 (8.5–23.0)		14.5 (8.3–20.4)		17.7 (11.3–25.8)		18.5 (10.5–26.0)	
	GA	9	14.1 (9.1–18.2)	0.57	13.5 (4.8–26.1)	0.82	18.1 (7.0–23.4)	0.64	22.2 (14.3–26.2)	0.43
	AA	–	–	–	–	–	–	–	–	–
	GA + AA	–	–	–	–	–	–	–	–	–
*XPG* Asn1104His (rs17655)	GG	41	15.9 (9.3–22.7)		13.5 (4.6–20.2)		19.4 (11.8–26.8)		18.7 (10.8–25.3)	
	GC	22	12.2 (5.8–17.1)		16.4 (10.8–23.7)		14.7 (11.2–24.5)		19.5 (10.5–33.3)	
	CC	5	15.9 (7.2–22.1)	0.24	9.1 (7.3–12.3)	0.17	16.3 (0.6–24.2)	0.23	19.1 (12.9–30.0)	0.71
	GC + CC	27	13.9 (5.8–18.5)	0.13	14.5 (9.1–21.6)	0.44	14.9 (10.7–24.2)	0.09	19.1 (10.5–31.7)	0.42

Associations with *p*-value < 0.05 are shown in bold.

#### Is protein activity related to level of gene transcription?

**Figure 6:**
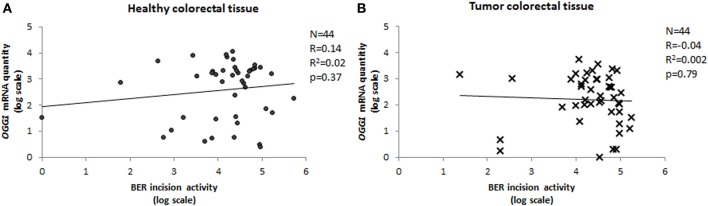
**Correlation between *OGG1* expression and BER incision activity**. Relative quantity of *OGG1* transcripts measured in 44 paired healthy **(A)** and tumor **(B)** tissue samples plotted against BER activity (Pearson's correlation coefficient).

We have measured the amount of OGG1 transcripts in paired tumor-healthy human colorectal tissues and compared it with the BER-related incision activity, which represents mainly OGG1 activity. The activity of the protein was completely independent of the mRNA quantity, with Pearson's correlation coefficient close to 0 for both tumor and normal tissue (Figure 6). Lack of a relationship between mRNA level and activity of the protein is not rare in the literature (Damia et al., 1998; Vogel et al., 2000; Paz-Elizur et al., 2007; Stevens et al., 2008; Slyskova et al., 2012a,b). On the contrary, there is growing evidence on the important role of regulation of enzyme activity at post-transcriptional and post-translational levels. *OGG1* is a housekeeping gene of constitutive expression independent of the cell cycle (Dhenaut et al., 2000). It might be regulated via two CpG islands located in the promoter region; however, this was not the case in our samples since none of 88 samples exhibited C methylation in *OGG1* promoter (Slyskova et al., 2012b). OGG1 has eight alternative isoforms/splicing variants of two major groups; type 1 acts in the nucleus, and type 2 in the mitochondria (Boiteux and Radicella, 2000). However, this would not serve as an explanation of missing mRNA quantity/protein activity correlation either, since all transcript variants have been covered in the assay. Nevertheless, other mechanisms might regulate gene activity; for example 160 microRNAs identified up to now are able to bind to OGG1 transcripts (http://bioinformatics.ekmd.huji.ac.il/reptar/gene_report.php?species=human&id=12458). Above all, two post-translational modifications—phosphorylation and nitrosylation—modulate the final protein activity. Another source of variability might be represented by protein–protein interactions (Fan and Wilson, 2005).

The mRNA expression of the majority of proteins forming the pre-incision complex of NER was also measured and plotted against the overall NER activity. The expression level of none of the 17 studied genes was significantly associated with the NER incision activity, except for *CDK7* protein involved in TFIIH complex in the tumor tissue only (Table 2). DNA damage recognition and incision is much more complex in NER as compared to BER. In BER, usually only 1 or 2 proteins are able to recognize and incise damage from DNA, while in NER, the whole complex of many proteins is required for lesion removal. NER proteins work in an interactive downstream manner and are known to be substantially regulated at a post-translational level, which makes the lack of correlation of single gene expression and endpoint NER incision activity understandable. According to our results and the results of other research groups (Damia et al., 1998; Vogel et al., 2000; Paz-Elizur et al., 2007; Stevens et al., 2008; Hanova et al., 2011; Slyskova et al., 2012b), expression analysis of single genes is not a sufficiently informative marker of activity of protein or protein complexes.

**Table 2:** **Correlation of expression of 17 genes involved in NER pre-incision complex with overall NER incision activity (Pearson's correlation coefficient)**.

**Gene**	**NER activity**			
	**Healthy tissue (*N* = 44)**		**Tumor (*N* = 44)**	
	***R***	***p*-value**	***R***	***p*-value**
*CCNH*	−0.146	0.35	−0.028	0.86
*CDK7*	−0.012	0.94	0.320	**0.036**
*CSB*	−0.101	0.51	−0.039	0.80
*DDB1*	−0.158	0.31	−0.094	0.54
*DDB2*	−0.200	0.19	−0.183	0.23
*ERCC1*	−0.133	0.39	−0.146	0.35
*LIG1*	0.086	0.58	0.123	0.43
*MNAT1*	−0.125	0.42	−0.094	0.54
*RAD23B*	−0.030	0.85	0.190	0.22
*RPA1*	−0.079	0.61	−0.05	0.75
*RPA2*	0.007	0.96	0.067	0.67
*RPA3*	−0.039	0.80	0.225	0.14
*XPA*	−0.168	0.28	−0.246	0.11
*XPB*	−0.055	0.73	−0.009	0.96
*XPC*	−0.062	0.69	−0.004	0.98
*XPD*	−0.164	0.29	−0.001	0.99
*XPF*	−0.136	0.38	−0.091	0.56

Associations with *p*-value < 0.05 are shown in bold.

## Concluding remarks

Analyzing DNA repair activity in target tissue might represent an important step toward individualized anti-cancer therapy. Previously we have shown that activities of the BER and NER pathways positively correlate between white blood cells and healthy colon tissue, but not between blood cells and tumor (Slyskova et al., 2012b). Therefore, methods for assessing functionality of DNA repair in solid tissues are warranted. New comet-based repair assays are reliable, simple, fast, and of low cost. An advanced medium-throughput format is suitable for large epidemiological studies. We have also shown that measuring DNA repair activity is not easily replaceable by a genomic or transcriptomic approach, but should be applied with the latter techniques in a complementary manner.

## Funding

This work was supported by CZ: GACR: GAP 304/12/1585. Sabine A. S. Langie was supported by the Centre for Brain Ageing and Vitality, which is funded through the Lifelong Health and Wellbeing cross council initiative by the MRC, BBSRC, EPSRC, and ESRC in the UK, and currently Sabine A. S. Langie is the beneficiary of a post-doctoral grant from the AXA Research Fund.

### Conflict of interest statement

The authors declare that the research was conducted in the absence of any commercial or financial relationships that could be construed as a potential conflict of interest.
